# Supplementary material for: Evolutionary stability of collateral sensitivity to antibiotics in the model pathogen Pseudomonas aeruginosa
Source: eLife. 2019 Oct 29;8:e51481. doi: 10.7554/eLife.51481 (PMC6881144; doi:10.7554/eLife.51481)
Supplement: Supplementary file 1. [file elife-51481-supp1.docx]

Figure 1–supplementary table 1. Antibiotic concentrations for evolution experiment.

| **Previously**  **evolved**  **resistant**  **population** | **New antibiotic** | | | **For maintenance of original resistance**^c^ |
| --- | --- | --- | --- | --- |
|  | **First dose (~IC_50_)** | **Final dose mild**^a^ | **Final dose strong**^b^ |  |
| CAR-10 | 410 ng/ml GEN | 570 ng/ml GEN | 890 ng/ml GEN | + 87 µg/ml CAR |
| GEN-4 | 1.0 µg/ml CAR | 30 µg/ml CAR | 87 µg/ml CAR | + 890 ng/ml GEN |
| PIT-1 | 2.2 µg/ml STR | 8.5 µg/ml STR | 21 µg/ml STR | + 4 µg/ml  PIT |
| STR-4 | 0.68 µg/ml PIT | 1.8 µg/ml PIT | 4 µg/ml  PIT | + 21 µg/ml  STR |

^a^ Approx. >IC_95_ of hypersensitive population specified in column 1

^b^ Approx. >IC_95_ of wild type PA14

^c^ Added to treatment groups mild+constrained, strong+constrained

Figure 3–supplementary table 1. Evaluation of the effect of the pace of dose increase (mild or strong) and evolutionary constraint (constrained or unconstrained) on cumulative relative growth^a^.

| **Antibiotic** | **Variable** | **χ^2^** | **Adjusted *P*** |
| --- | --- | --- | --- |
| GEN | Pace | 14.7 | 0.0002 |
|  | Constraint | 158.1 | <0.0001 |
| CAR | Pace | 18.1 | <0.0001 |
|  | Constraint | 53.8 | <0.0001 |

^a^ Separate GLMs were performed for each antibiotic and with the cumulative relative growth of surviving populations as the response variable, and pace of dose increase (strong or mild) and constraint (constrained or unconstrained) as explanatory fixed factors. Starting clonal population was considered as a nested random factor. We used a type-II Wald χ^2^-test to evaluate the effect of these variables. We used the false discovery rate to adjust the *P* values for multiple comparisons.

Figure 3–supplementary table 2. Evaluation of the changes in resistance^a^.

| **Resistant to** | **Challenged with** | **Treatment** | **Number of populations** | **Adjusted *P*** |
| --- | --- | --- | --- | --- |
| CAR | CAR | no drug | 8 | 0.94485 |
| CAR | CAR | strong | 8 | 0.7736 |
| CAR | CAR | strong+constrained | 8 | 0.96151 |
| CAR | CAR | mild | 8 | 0.6425 |
| CAR | CAR | mild+constrained | 8 | 0.29516 |
| CAR | GEN | no drug | 8 | 0.72291 |
| CAR | GEN | strong | 8 | 0.00013 |
| CAR | GEN | strong+constrained | 8 | 0.00489 |
| CAR | GEN | mild | 8 | 0.00124 |
| CAR | GEN | mild+constrained | 8 | 0.0016 |
| GEN | CAR | no drug | 8 | <0.00001 |
| GEN | CAR | strong | 7 | <0.00001 |
| GEN | CAR | strong+constrained | 2 | 0.10237 |
| GEN | CAR | mild | 8 | <0.00001 |
| GEN | CAR | mild+constrained | 8 | <0.00001 |
| GEN | GEN | no drug | 8 | 0.03872 |
| GEN | GEN | strong | 7 | 0.00065 |
| GEN | GEN | strong+constrained | 2 | 0.61634 |
| GEN | GEN | mild | 8 | <0.00001 |
| GEN | GEN | mild+constrained | 8 | 0.00102 |

^a^ *P* values were obtained from a series of Student’s t-tests per treatment for populations with ancestral resistance against a given antibiotic and evaluated against two drugs. We used the false discovery rate correction method to adjust *P* values for multiple comparisons.

**Figure 3–supplementary table 3. Fold change IC_90_ of evolved populations, relative to their IC_90_ at the beginning of the experiment.**

| **Resistant to** | **Population** | **Plate** | **Clone** | **Challenged with** | **Treatment** | **Fold-IC_90_** |
| --- | --- | --- | --- | --- | --- | --- |
| CAR | 10 | a | 1 | GEN | constrained+strong | 5 |
|  |  |  |  |  | constrained+mild | 5 |
|  |  |  |  |  | strong | 9 |
|  |  |  |  |  | mild | 5 |
|  |  |  | 2 | GEN | constrained+strong | 9 |
|  |  |  |  |  | constrained+mild | 5 |
|  |  |  |  |  | strong | 9 |
|  |  |  |  |  | mild | 5 |
|  |  |  | 3 | GEN | constrained+strong | 5 |
|  |  |  |  |  | constrained+mild | 5 |
|  |  |  |  |  | strong | 9 |
|  |  |  |  |  | mild | 9 |
|  |  |  | 4 | GEN | constrained+strong | 5 |
|  |  |  |  |  | constrained+mild | 2 |
|  |  |  |  |  | strong | 1.5 |
|  |  |  |  |  | mild | 2 |
|  |  | b | 1 | GEN | constrained+strong | >9 |
|  |  |  |  |  | constrained+mild | 5 |
|  |  |  |  |  | strong | >9 |
|  |  |  |  |  | mild | 9 |
|  |  |  | 2 | GEN | constrained+strong | >9 |
|  |  |  |  |  | constrained+mild | >9 |
|  |  |  |  |  | strong | >9 |
|  |  |  |  |  | mild | 9 |
|  |  |  | 3 | GEN | constrained+strong | >9 |
|  |  |  |  |  | constrained+mild | >9 |
|  |  |  |  |  | strong | 9 |
|  |  |  |  |  | mild | >9 |
|  |  |  | 4 | GEN | constrained+strong | 9 |
|  |  |  |  |  | constrained+mild | >9 |
|  |  |  |  |  | strong | >9 |
|  |  |  |  |  | mild | 9 |
| GEN | 4 | a | 1 | CAR | constrained+strong | 48 |
|  |  |  |  |  | constrained+mild | 12 |
|  |  |  |  |  | strong | 48 |
|  |  |  |  |  | mild | 12 |
|  |  |  | 2 | CAR | constrained+strong | 48 |
|  |  |  |  |  | constrained+mild | 24 |
|  |  |  |  |  | strong | extinct |
|  |  |  |  |  | mild | 12 |
|  |  |  | 3 | CAR | constrained+strong | 48 |
|  |  |  |  |  | constrained+mild | 24 |
|  |  |  |  |  | strong | extinct |
|  |  |  |  |  | mild | 12 |
|  |  |  | 4 | CAR | constrained+strong | 48 |
|  |  |  |  |  | constrained+mild | 48 |
|  |  |  |  |  | strong | extinct |
|  |  |  |  |  | mild | 48 |
|  |  | b | 1 | CAR | constrained+strong | extinct |
|  |  |  |  |  | constrained+mild | 24 |
|  |  |  |  |  | strong | extinct |
|  |  |  |  |  | mild | 24 |
|  |  |  | 2 | CAR | constrained+strong | 24 |
|  |  |  |  |  | constrained+mild | 24 |
|  |  |  |  |  | strong | extinct |
|  |  |  |  |  | mild | 24 |
|  |  |  | 3 | CAR | constrained+strong | 24 |
|  |  |  |  |  | constrained+mild | 24 |
|  |  |  |  |  | strong | 48 |
|  |  |  |  |  | mild | 24 |
|  |  |  | 4 | CAR | constrained+strong | 48 |
|  |  |  |  |  | constrained+mild | 48 |
|  |  |  |  |  | strong | extinct |
|  |  |  |  |  | mild | 24 |
| PIT | 1 | a | 1 | STR | constrained+strong | extinct |
|  |  |  |  |  | constrained+mild | >32 |
|  |  |  |  |  | strong | extinct |
|  |  |  |  |  | mild | extinct |
|  |  |  | 2 | STR | constrained+strong | extinct |
|  |  |  |  |  | constrained+mild | 8 |
|  |  |  |  |  | strong | 16 |
|  |  |  |  |  | mild | 32 |
|  |  |  | 3 | STR | constrained+strong | 32 |
|  |  |  |  |  | constrained+mild | 8 |
|  |  |  |  |  | strong | extinct |
|  |  |  |  |  | mild | extinct |
|  |  |  | 4 | STR | constrained+strong | extinct |
|  |  |  |  |  | constrained+mild | 8 |
|  |  |  |  |  | strong | extinct |
|  |  |  |  |  | mild | extinct |
|  |  | b | 1 | STR | constrained+strong | extinct |
|  |  |  |  |  | constrained+mild | >32 |
|  |  |  |  |  | strong | extinct |
|  |  |  |  |  | mild | extinct |
|  |  |  | 2 | STR | constrained+strong | extinct |
|  |  |  |  |  | constrained+mild | 16 |
|  |  |  |  |  | strong | 16 |
|  |  |  |  |  | mild | 32 |
|  |  |  | 3 | STR | constrained+strong | >32 |
|  |  |  |  |  | constrained+mild | 16 |
|  |  |  |  |  | strong | extinct |
|  |  |  |  |  | mild | extinct |
|  |  |  | 4 | STR | constrained+strong | extinct |
|  |  |  |  |  | constrained+mild | 8 |
|  |  |  |  |  | strong | extinct |
|  |  |  |  |  | mild | 32 |
| STR | 4 | a | 1 | STR | constrained+strong | extinct |
|  |  |  |  |  | constrained+mild | 7 |
|  |  |  |  |  | strong | extinct |
|  |  |  |  |  | mild | 7 |
|  |  |  | 2 | STR | constrained+strong | extinct |
|  |  |  |  |  | constrained+mild | extinct |
|  |  |  |  |  | strong | extinct |
|  |  |  |  |  | mild | extinct |
|  |  |  | 3 | STR | constrained+strong | 7 |
|  |  |  |  |  | constrained+mild | 7 |
|  |  |  |  |  | strong | extinct |
|  |  |  |  |  | mild | extinct |
|  |  |  | 4 | STR | constrained+strong | 14 |
|  |  |  |  |  | constrained+mild | 14 |
|  |  |  |  |  | strong | 7 |
|  |  |  |  |  | mild | 14 |
|  |  | b | 1 | STR | constrained+strong | 7 |
|  |  |  |  |  | constrained+mild | 7 |
|  |  |  |  |  | strong | extinct |
|  |  |  |  |  | mild | extinct |
|  |  |  | 2 | STR | constrained+strong | extinct |
|  |  |  |  |  | constrained+mild | extinct |
|  |  |  |  |  | strong | extinct |
|  |  |  |  |  | mild | 7 |
|  |  |  | 3 | STR | constrained+strong | extinct |
|  |  |  |  |  | constrained+mild | 7 |
|  |  |  |  |  | strong | extinct |
|  |  |  |  |  | mild | extinct |
|  |  |  | 4 | STR | constrained+strong | 14 |
|  |  |  |  |  | constrained+mild | 4 |
|  |  |  |  |  | strong | 14 |
|  |  |  |  |  | mild | 14 |

**Figure 5-supplementary table 1. Evaluation of the changes in resistance for surviving populations in the generalization experiment^a^.**

| **Resistant to** | **Challenged with** | **Treatment** | **Adjusted *P*** |
| --- | --- | --- | --- |
| CAR | CAR | constrained | 0.2872 |
| CAR | CAR | strong | 0.0002 |
| CAR | GEN | constrained | 0.2872 |
| CAR | GEN | strong | 0.0015 |
| CAR | CAR | constrained | 0.989 |
| CAR | CAR | strong | 0.1424 |
| CAR | STR | constrained | 0.0002 |
| CAR | STR | strong | 0.0001 |
| CEF | CEF | constrained | 0.0563 |
| CEF | CEF | strong | 0.1424 |
| CEF | GEN | constrained | 0.0563 |
| CEF | GEN | strong | 0.0015 |
| CEF | CEF | constrained | 0.5278 |
| CEF | CEF | strong | 0.5161 |
| CEF | STR | constrained | 0.0002 |
| CEF | STR | strong | 0.0001 |
| CIP | CAR | constrained | 0.2872 |
| CIP | CAR | strong | 0.0088 |
| CIP | CIP | constrained | 0.2872 |
| CIP | CIP | strong | 0.2872 |
| CIP | CIP | constrained | 0.1424 |
| CIP | CIP | strong | 0.0001 |
| CIP | GEN | constrained | 0.0004 |
| CIP | GEN | strong | 0.0001 |
| CIP | CIP | constrained | 1 |
| CIP | CIP | strong | 0.0002 |
| CIP | PIT | constrained | 0.2872 |
| CIP | PIT | strong | 0.0002 |
| CIP | CIP | constrained | 0.0001 |
| CIP | CIP | strong | 1 |
| CIP | STR | constrained | 0.0001 |
| CIP | STR | strong | 0.0001 |
| GEN | CAR | constrained | 0.0088 |
| GEN | CAR | strong | 0.0002 |
| GEN | GEN | constrained | 0.0169 |
| GEN | GEN | strong | 0.0022 |
| GEN | GEN | constrained | 0.0563 |
| GEN | GEN | strong | 0.0563 |
| GEN | PIT | constrained | 0.0563 |
| GEN | PIT | strong | 0.4922 |
| PIT | PIT | constrained | 0.2872 |
| PIT | PIT | strong | 0.2872 |
| PIT | GEN | constrained | 0.0001 |
| PIT | GEN | strong | 0.0017 |
| PIT | PIT | constrained | 0.2872 |
| PIT | PIT | strong | 1 |
| PIT | STR | constrained | 0.2872 |
| PIT | STR | strong | 0.0923 |
| STR | CAR | constrained | 0.0088 |
| STR | CAR | strong | 0.0002 |
| STR | STR | constrained | 0.068 |
| STR | STR | strong | 0.0022 |
| STR | PIT | constrained | extinct |
| STR | PIT | strong | 0.2872 |

*^a^ P* values were obtained from a series of Student’s t-tests per treatment for populations with ancestral resistance against a given antibiotic and evaluated against two or four drugs. We used the false discovery rate correction method to adjust *P* values for multiple comparisons.

**Figure 5-supplementary table 2. Fold change IC_90_ of evolved populations, relative to their IC_90_ at the beginning of the experiment.**

| **Resistant to** | **Population** | **Challenged with** | **Treatment** | **Fold-IC_90_** |
| --- | --- | --- | --- | --- |
| CIP | 2 | CAR | constrained | extinct |
|  |  |  | strong | >18 |
|  |  | GEN | constrained | 3 |
|  |  |  | strong | 6 |
|  |  | PIT | constrained | 8 |
|  |  |  | strong | 8 |
|  |  | STR | constrained | 7 |
|  |  |  | strong | 7 |
| CIP | 3 | CAR | constrained | extinct |
|  |  |  | strong | 11 |
|  |  | GEN | constrained | 6 |
|  |  |  | strong | 6 |
|  |  | PIT | constrained | extinct |
|  |  |  | strong | 10 |
|  |  | STR | constrained | 8 |
|  |  |  | strong | 8 |
| CIP | 5 | CAR | constrained | extinct |
|  |  |  | strong | extinct |
|  |  | GEN | constrained | 4 |
|  |  |  | strong | 2 |
|  |  | PIT | constrained | extinct |
|  |  |  | strong | 40 |
|  |  | STR | constrained | 19 |
|  |  |  | strong | 10 |
| CIP | 7 | CAR | constrained | extinct |
|  |  |  | strong | 16 |
|  |  | GEN | constrained | 9 |
|  |  |  | strong | 9 |
|  |  | PIT | constrained | extinct |
|  |  |  | strong | 20 |
|  |  | STR | constrained | >24 |
|  |  |  | strong | 12 |
| CIP | 9 | CAR | constrained | extinct |
|  |  |  | strong | extinct |
|  |  | GEN | constrained | 13 |
|  |  |  | strong | 7 |
|  |  | PIT | constrained | extinct |
|  |  |  | strong | extinct |
|  |  | STR | constrained | 16 |
|  |  |  | strong | 32 |
| CIP | 10 | CAR | constrained | 26 |
|  |  |  | strong | extinct |
|  |  | GEN | constrained | 5 |
|  |  |  | strong | 10 |
|  |  | PIT | constrained | extinct |
|  |  |  | strong | 7 |
|  |  | STR | constrained | 7 |
|  |  |  | strong | 7 |
| CEF | 2 | GEN | constrained | extinct |
|  |  |  | strong | extinct |
|  |  | STR | constrained | 14 |
|  |  |  | strong | 14 |
| CEF | 3 | GEN | constrained | extinct |
|  |  |  | strong | extinct |
|  |  | STR | constrained | 16 |
|  |  |  | strong | 32 |
| CEF | 7 | GEN | constrained | extinct |
|  |  |  | strong | 11 |
|  |  | STR | constrained | 7 |
|  |  |  | strong | 14 |
| CEF | 8 | GEN | constrained | >13 |
|  |  |  | strong | 3 |
|  |  | STR | constrained | 7 |
|  |  |  | strong | 3 |
| CEF | 9 | GEN | constrained | extinct |
|  |  |  | strong | 27 |
|  |  | STR | constrained | extinct |
|  |  |  | strong | 7 |
| CEF | 10 | GEN | constrained | 13 |
|  |  |  | strong | 6 |
|  |  | STR | constrained | 11 |
|  |  |  | strong | 5 |
| CAR | 1 | GEN | constrained | not tested |
|  |  |  | strong | not tested |
|  |  | STR | constrained | extinct |
|  |  |  | strong | 8 |
| CAR | 2 | GEN | constrained | 6 |
|  |  |  | strong | 2 |
|  |  | STR | constrained | not tested |
|  |  |  | strong | not tested |
| CAR | 3 | GEN | constrained | extinct |
|  |  |  | strong | extinct |
|  |  | STR | constrained | 24 |
|  |  |  | strong | 6 |
| CAR | 4 | GEN | constrained | extinct |
|  |  |  | strong | extinct |
|  |  | STR | constrained | 7 |
|  |  |  | strong | 14 |
| CAR | 5 | GEN | constrained | extinct |
|  |  |  | strong | >11 |
|  |  | STR | constrained | 6 |
|  |  |  | strong | 12 |
| CAR | 8 | GEN | constrained | extinct |
|  |  |  | strong | 6 |
|  |  | STR | constrained | >16 |
|  |  |  | strong | 8 |
| CAR | 10 | GEN | constrained | extinct |
|  |  |  | strong | 5 |
|  |  | STR | constrained | 24 |
|  |  |  | strong | 6 |
| PIT | 1 | GEN | constrained | extinct |
|  |  |  | strong | 17 |
|  |  | STR | constrained | extinct |
|  |  |  | strong | extinct |
| PIT | 2 | GEN | constrained | extinct |
|  |  |  | strong | 3 |
|  |  | STR | constrained | not-tested |
|  |  |  | strong | not-tested |
| PIT | 3 | GEN | constrained | extinct |
|  |  |  | strong | 9 |
|  |  | STR | constrained | extinct |
|  |  |  | strong | extinct |
| PIT | 5 | GEN | constrained | extinct |
|  |  |  | strong | 4 |
|  |  | STR | constrained | extinct |
|  |  |  | strong | >14 |
| PIT | 6 | GEN | constrained | extinct |
|  |  |  | strong | 13 |
|  |  | STR | constrained | 7 |
|  |  |  | strong | 14 |
| PIT | 9 | GEN | constrained | extinct |
|  |  |  | strong | 3 |
|  |  | STR | constrained | extinct |
|  |  |  | strong | 2 |
| PIT | 10 | GEN | constrained | not tested |
|  |  |  | strong | not tested |
|  |  | STR | constrained | extinct |
|  |  |  | strong | extinct |
| GEN | 4 | CAR | constrained | extinct |
|  |  |  | strong | extinct |
|  |  | PIT | constrained | 4 |
|  |  |  | strong | extinct |
| GEN | 5 | CAR | constrained | >64 |
|  |  |  | strong | >64 |
|  |  | PIT | constrained | extinct |
|  |  |  | strong | 2 |
| GEN | 6 | CAR | constrained | >43 |
|  |  |  | strong | 21 |
|  |  | PIT | constrained | extinct |
|  |  |  | strong | 2 |
| GEN | 8 | CAR | constrained | 64 |
|  |  |  | strong | >64 |
|  |  | PIT | constrained | extinct |
|  |  |  | strong | extinct |
| GEN | 9 | CAR | constrained | extinct |
|  |  |  | strong | 64 |
|  |  | PIT | constrained | 23 |
|  |  |  | strong | extinct |
| GEN | 10 | CAR | constrained | extinct |
|  |  |  | strong | 32 |
|  |  | PIT | constrained | extinct |
|  |  |  | strong | extinct |
| STR | 2 | CAR | constrained | >21 |
|  |  |  | strong | 11 |
|  |  | PIT | constrained | not tested |
|  |  |  | strong | not tested |
| STR | 3 | CAR | constrained | >32 |
|  |  |  | strong | extinct |
|  |  | PIT | constrained | not tested |
|  |  |  | strong | not tested |
| STR | 4 | CAR | constrained | extinct |
|  |  |  | strong | >21 |
|  |  | PIT | constrained | extinct |
|  |  |  | strong | 20 |
| STR | 6 | CAR | constrained | >32 |
|  |  |  | strong | >32 |
|  |  | PIT | constrained | not tested |
|  |  |  | strong | not tested |
| STR | 8 | CAR | constrained | extinct |
|  |  |  | strong | >32 |
|  |  | PIT | constrained | not tested |
|  |  |  | strong | not tested |
| STR | 10 | CAR | constrained | extinct |
|  |  |  | strong | 21 |
|  |  | PIT | constrained | not tested |
|  |  |  | strong | not tested |

**Figure 6–supplementary table 1. Analysis of predictors of extinction during antibiotic switches in the generalized evolution experiment using GLM.**

| Measure of CS effects | Fold-IC_75_ | | Fold-IC_90_ | | Relative AUC | |
| --- | --- | --- | --- | --- | --- | --- |
| Predictor | *F* value | *P* value^a^ | *F* value | *P* value^a^ | *F* value | *P* value^a^ |
| Target of drug A | 0.9421 | 0.42913 | 0.9279 | 0.43409 | 0.9265 | 0.43458 |
| Target of drug B | 11.0163 | **0.01055** | 10.8507 | **0.01096** | 10.8342 | **0.01100** |
| Average CS effect size | 2.4268 | 0.15789 | 2.8476 | 0.12999 | 1.0735 | 0.33046 |
| Average growth rate | 3.0251 | 0.12018 | 2.4020 | 0.15977 | 4.1560 | 0.07583 |

*^a^* Significant associations are indicated in bold font (*P* < 0.05).

**Figure 6–supplementary table 2. Analysis of predictors of collateral sensitivity stability in the unconstrained treatment group (‘strong’) in the generalized evolution experiment using GLM.**

Part 1. Changes in resistance to drug A

| Measure of CS effects | Fold-IC_75_ | | Fold-IC_90_ | | Relative AUC | |
| --- | --- | --- | --- | --- | --- | --- |
| Predictor | *F* value | *P* value^a^ | *F* value | *P* value^a^ | *F* value | *P* value^a^ |
| Target of drug A | 15.2664 | **0.0019** | 18.2714 | **0.0010** | 14.1906 | **0.0023** |
| Target of drug B | 0.3053 | 0.5957 | 0.3654 | 0.5623 | 0.2838 | 0.6087 |
| Average CS effect size | 15.9060 | **0.0040** | 22.8112 | **0.0014** | 24.7715 | **0.0011** |
| Average growth rate | 18.3780 | **0.0027** | 19.7958 | **0.0021** | 6.5329 | **0.0339** |

Part 2. Changes in resistance to drug B

| Measure of CS effects | Fold-IC_75_ | | Fold-IC_90_ | | Relative AUC | |
| --- | --- | --- | --- | --- | --- | --- |
| Predictor | *F* value | *P* value^a^ | *F* value | *P* value^a^ | *F* value | *P* value^a^ |
| Target of drug A | 0.0866 | 0.9179 | 0.2099 | 0.8150 | 0.0755 | 0.9280 |
| Target of drug B | 0.1721 | 0.6892 | 0.4170 | 0.5365 | 0.1499 | 0.7087 |
| Average CS effect size | 6.7087 | **0.0321** | 25.2151 | **0.0010** | 1.2987 | 0.2874 |
| Average growth rate | 7.8961 | **0.0228** | 21.5674 | **0.0017** | 10.4001 | **0.0122** |

*^a^* Significant associations are indicated in bold font (*P* < 0.05).
