# Supplementary material for: Evolutionary stability of collateral sensitivity to antibiotics in the model pathogen Pseudomonas aeruginosa
Source: eLife. 2019 Oct 29;8:e51481. doi: 10.7554/eLife.51481 (PMC6881144; doi:10.7554/eLife.51481)
Supplement: Transparent reporting form [file elife-51481-transrepform.docx]

***eLife’s* transparent reporting form**

We encourage authors to provide detailed information *within their submission* to facilitate the interpretation and replication of experiments. Authors can upload supporting documentation to indicate the use of appropriate reporting guidelines for health-related research (see [EQUATOR Network](http://www.equator-network.org/%20)), life science research (see the [BioSharing Information Resource](https://biosharing.org/)), or the [ARRIVE guidelines](http://www.plosbiology.org/article/info:doi/10.1371/journal.pbio.1000412) for reporting work involving animal research. Where applicable, authors should refer to any relevant reporting standards documents in this form.

If you have any questions, please consult our Journal Policies and/or contact us: [editorial@elifesciences.org](mailto:editorial@elifesciences.org).

**Sample-size estimation**

- You should state whether an appropriate sample size was computed when the study was being designed
- You should state the statistical method of sample size computation and any required assumptions
- If no explicit power analysis was used, you should describe how you decided what sample (replicate) size (number) to use

Please outline where this information can be found within the submission (e.g., sections or figure legends), or explain why this information doesn’t apply to your submission:

We performed 2 sets of evolution experiments:

For the first evolution experiment, we considered four antibiotic sequences (Drug A -> Drug B), each with five experimental conditions. Given these, we included the maximum number of biological replicates per antibiotic pair and experimental conditions fitting in a 96-well microtiter plate; yielding a total of four biological replicates. We duplicated the obtained 96-well plate and ran it in parallel during experimental evolution. In subsequent experiments we took the evolved populations from both plates and independently considered 3 technical replicates per experimental condition.

For the second experiment, we considered 14 antibiotic sequences, each with 3 experimental conditions and including a total of 38 biological replicates. This number fits into 3 randomized 96-well microtiter plates (Please see details in the Materials and Methods section). In subsequent experiments we took the surviving evolved populations from both plates and independently considered 3 technical replicates per experimental condition.

Further details can be found in figure legends or the Materials and Methods.

**Replicates**

- You should report how often each experiment was performed
- You should include a definition of biological versus technical replication
- The data obtained should be provided and sufficient information should be provided to indicate the number of independent biological and/or technical replicates
- If you encountered any outliers, you should describe how these were handled
- Criteria for exclusion/inclusion of data should be clearly stated
- High-throughput sequence data should be uploaded before submission, with a private link for reviewers provided (these are available from both GEO and ArrayExpress)

Please outline where this information can be found within the submission (e.g., sections or figure legends), or explain why this information doesn’t apply to your submission:

We performed two sets of evolution experiments.

For the first set, we considered four biological replicates in duplicate for the main evolution experiments. In subsequent experiments, each of the evolved populations was analyzed in triplicate.

For the second set, we considered a total of 38 independent biological replicates for the main evolution experiment. In subsequent experiments, each of the evolved populations was analyzed in triplicate.

All the replicate numbers are given in figure legends and/or supplementary figures.

**Statistical reporting**

- Statistical analysis methods should be described and justified
- Raw data should be presented in figures whenever informative to do so (typically when N per group is less than 10)
- For each experiment, you should identify the statistical tests used, exact values of N, definitions of center, methods of multiple test correction, and dispersion and precision measures (e.g., mean, median, SD, SEM, confidence intervals; and, for the major substantive results, a measure of effect size (e.g., Pearson's r, Cohen's d)
- Report exact p-values wherever possible alongside the summary statistics and 95% confidence intervals. These should be reported for all key questions and not only when the p-value is less than 0.05.

Please outline where this information can be found within the submission (e.g., sections or figure legends), or explain why this information doesn’t apply to your submission:

All statistical tests, replicate numbers, and precision measures are indicated in the legends of all figures, or are summarized in supplementary tables.

(For large datasets, or papers with a very large number of statistical tests, you may upload a single table file with tests, Ns, etc., with reference to sections in the manuscript.)

**Group allocation**

- Indicate how samples were allocated into experimental groups (in the case of clinical studies, please specify allocation to treatment method); if randomization was used, please also state if restricted randomization was applied
- Indicate if masking was used during group allocation, data collection and/or data analysis

Please outline where this information can be found within the submission (e.g., sections or figure legends), or explain why this information doesn’t apply to your submission:

The different groups of treatments and antibiotics were always distributed in fully randomized order across 96-well microtiter plates.

**Additional data files (“source data”)**

- We encourage you to upload relevant additional data files, such as numerical data that are represented as a graph in a figure, or as a summary table
- Where provided, these should be in the most useful format, and they can be uploaded as “Source data” files linked to a main figure or table
- Include model definition files including the full list of parameters used
- Include code used for data analysis (e.g., R, MatLab)
- Avoid stating that data files are “available upon request”

Please indicate the figures or tables for which source data files have been provided:

We include 11 Source Data files which contain the data used for the generation of all the figures as well as the genomic data.
